# Chronic disease multimorbidity among the Canadian population: prevalence and associated lifestyle factors

**DOI:** 10.1186/s13690-021-00583-7

**Published:** 2021-04-28

**Authors:** Nigatu Regassa Geda, Bonnie Janzen, Punam Pahwa

**Affiliations:** 1 College of Development Studies, Center for Population Studies, Addis Ababa University, P.o.box 1176, Addis Ababa, Ethiopia; 2 Frontieri Consult PLC, Water Hygiene and Sanitation, Addis Ababa, Ethiopia; 3 Dept of Community Health & Epidemiology, Collège of Medicine, University of Saskatchewan, Saskatoon, Canada; 4 Canadian Centre for Health and Safety in Agriculture, University of Saskatchewan, Saskatoon, Canada

**Keywords:** Chronic disease, Morbidity, Multimorbidity, Lifestyle factors, Canada

## Abstract

**Background:**

Chronic diseases is increasingly becoming one of the most pressing public health concerns in most part of the world, including the Canadian population. The purpose of this study was to estimate the prevalence of multimorbidity in the general population based on 14 major chronic diseases and examine associations with lifestyle/behavioral factors.

**Methods:**

The data source was the 2015–2016 Canadian Community Health Survey (CCHS). The CCHS is a cross sectional, complex multi-stage survey based on information collected from 109,659 participants aged 12+, covering all provinces and territories. Multimorbidity was defined as the co-occurrence of two or more chronic diseases within a person. Multiple logistic regression was used to examine the key determinants of multimorbidity.

**Results:**

The prevalence of multimorbidity was 33 %. Adjusting for sociodemographic variables, there was an increased odd of multimorbidity for those having a sedentary lifestyle (AOR = 1.06; CI:1.01–1.11) and being obese (AOR = 1.37; CI:1.32–1.43) or overweight (AOR = 2.65; CI: 2.54–2.76). There were two statistically significant interactions, between sex and smoking, and between immigration status and alcohol intake. Smoking was more strongly associated with multimorbidity in females than males. The association between alcohol intake and multimorbidity was also dependent upon immigration status.

**Conclusions:**

Given the high prevalence of multimorbidity among the general Canadian population, policy makers and service providers should give more attention to the behavioral/lifestyle factors which significantly predicted multimorbidity. Policy and program efforts that promote a healthy lifestyle should be a priority.

## Background

There has been a rapid rise in the prevalence of people living with multiple chronic health problems (i.e. multimorbidity)[1–4], including Canada[5, 6]. Although there is little consensus on how multimorbidity is conceptualized[7], most recent studies have defined the term as the co-occurrence of two or more (chronic) diseases within a person[8]. Multimorbidity is associated with significant reductions in functional status and quality of life[9, 10].

The prevalence of multimorbidity varies according to the definition used[2, 11]. In Western countries, the prevalence of multimorbidity has ranged between 20 and 30 % when the whole population was considered, and between 55 and 98 % when only older persons were included[12]. Multimorbidity prevalence estimates within Canada vary from 4 % to over 90 % due to dissimilar study designs and definitions of multimorbidity[6, 13–16].For example, a recent study[5] using administrative health data from seven provinces and three territories, estimated a prevalence of 26.5 % based on five conditions (cardiovascular disease, respiratory diseases, mental illness, hypertension, diabetes). Nicholson (2016) reported a prevalence of 42.6 % for the national population aged 18 years and over based on electronic medical record data[17]. In another study, Roberts et al. (2015) estimated the national prevalence of 12.9 % for two or more chronic diseases for all age groups based on data from the Canadian Community Health Survey[6].

Previous studies around the world have examined the key predictors of multimorbidity. Most of these studies have focused on sociodemographic factors and suggest an increased prevalence of multimorbidity with increasing age[6, 16], lower income[18, 19], and less education[18, 20, 21]. The few studies which have examined associations between lifestyle factors and multimorbidity suggest that the likelihood of multimorbidity increases with a more sedentary lifestyle ([22, 23], obesity[24], and lower alcohol intake [25–27]. Most Canadian studies have reported mixed results regarding the socioeconomic and demographic risk factors for multimorbidity [6, 7, 14–16], with relatively little attention paid to lifestyle factors[16].

Despite the increasing prevalence of multimorbidity over time in Canada, little attention has been given to this issue in the health care system. The very few population-based studies conducted in Canada have focused on a limited number of chronic diseases[5, 6] or potential correlates of multimorbidity[14], and/or investigated only demographic or geographic subpopulations[14, 15, 28]. To address this limitation, the present study has considered a more comprehensive list of chronic conditions, all ages, and all provinces of Canada. Drawing from the existing literature, we hypothesized that the prevalence of multimorbidity would be significantly higher among those who engage in riskier health behaviors (such as smoking, frequent alcohol consumption, a sedentary lifestyle, and/or having a higher BM).

## Data sources and methodology

### Data source, study design and study population

The present study is based on public use data from the 2015–2016 Canadian Community Health Survey (CCHS). The CCHS is a multistage complex cross-sectional survey that collects a wide range of health and sociodemographic information from participants [29]. The 2015–2016 CCHS data covered the population 12 years of age and over living in the ten provinces and the three territories and included a total of 109,659 respondents [29]. The data collection excluded persons living on reserves and other Aboriginal settlements in the provinces; full-time members of the Canadian Forces; the institutionalized population, children aged 12–17 living in foster care, and some remote areas[29]. The exclusions represent less than 3 % of the Canadian population aged 12 and over. The detailed description of methods, design, instruments, participants and sampling frame has previously been published by Statistics Canada:http://www23.statcan.gc.ca/imdb/p2SV.pl?Function=getSurvey&SDDS=3226.

### Study variables

The dependent variable, multimorbidity, was defined as having two or more (2+) chronic conditions from a list of fourteen self-reported diseases diagnosed by a health professional during a reference period of 6 months prior to the survey[29]. A recent Canadian study has suggested that limiting the conditions to fewer than seven chronic diseases may result in underestimation of the multimorbidity prevalence, and recommend including 12 or more chronic diseases[7]. The 14 diseases were selected for this study based on their severity, potential impact on mortality, and availability of the information in the CCHS data[29]. These were: joint pain, asthma, chronic obstructive pulmonary disease, sleep apnea, scoliosis, fibromyalgia, arthritis, osteoporosis, high blood pressure, heart disease, stroke, diabetes, cancer and mood disorder (i.e. depression, bipolar, mania, dysthymia). The multimorbidity variable was computed using simple counts of the reported chronic diseases. A dichotomous variable was created, with a code of “1” given if the respondent reported two or more chronic conditions and “0” if the respondent reported less than two conditions.

The explanatory variables were categorized into two major groups: sociodemographic characteristics (sex/gender, age, marital status living arrangement, ethnicity, immigration status, work status, education level, and income); and behavioral risk factors (physical activity level, smoking, alcohol intake, and body mass index (BMI)).

Regarding sociodemographic, age was classified into four groups (years):<24, 25–40, 41–65 and 65+. Ethnicity was grouped as white, aboriginal, or visible minority. Marital status included four categories: married, common-law, widowed/divorced/separated, and single/never married. Respondents’ educational attainment was grouped into three categories: less than secondary school graduation, secondary school graduation/no post-secondary education, and post-secondary certificate diploma/university education. Annual household income consisted of five groups, with $20,000 increments beginning with zero.: No income or less than $20,000, $20,000 to $39,999, $40,000 to $59,999, $60,000 to $79,999, and $80,000 or more. Work status was measured as ‘yes/no’ for a reference period of 12 months prior to the survey date. Work stress is a self-reported variable (i.e., respondents were asked if they currently experience work related stress) and was measure as ‘yes/no’. The variable ‘living arrangement’ was used as a proxy for presence/ absence of social support and was categorized as ‘living alone or living with family members/others’.

Among the lifestyle variables, smoking status was divided into three categories: daily, occasionally, and not at all. Physical activity was classified according to World Health Organization (WHO) recommendations (active, moderately active, mild active, sedentary)[30]. The WHO classification provides age dependent list of moderate physical activities with the corresponding minimum amount of time (in minutes per week) required for health life. BMI was calculated based on self-reported weight and height and categorized into obese (BMI 30.0 kg/m^2^), overweight (BMI 25.0–29.9 kg/m^2^) and normal (18.5–24.9 kg/m^2^). Alcohol intake was classified as regular, occasional, and never taker during the last 12 months.

### Statistical Analysis

Data management was carried out using STATA version 13 [31]. The distribution of multimorbidity across various sociodemographic and lifestyle/behavioral variables were described using frequencies and proportions.

The prevalence of multimorbidity was estimated for the entire sample using weighted proportions with 95 % confidence intervals (95 % CIs). Bivariate logistic regression analyses were conducted to see associations between an explanatory variable and multimorbidity. Variables with a p < 0.20 were selected for the multivariable logistic regression[29]. Multicollinearity among the explanatory variables was checked using the Variance Inflation Factor (VIF) and removed those with VIF greater than 2.5[29]. Odds ratios with 95 % confidence intervals were calculated for each factor in logistic regression model. Two-way interactions were assessed by entering the product of two hypothesized variables.

All analysis were weighted to ensure that the final estimates were representative of the Canadian population [29]. As CCHS employed cross sectional design, stratification and clustering effects were accounted for by computing robust standard errors of regression coefficients using Tylor linearization techniques.

## Results

Table 1 displays characteristics of the study participants(weighted). Overall, nearly one-half of the participants were males. The reported ethnicity for the vast majority was white. The largest proportion of participants were age 41–65 years (40.3 %), followed by those in the 25-40-year age group (23.8 %). Over half of the respondents were married or cohabitating (58 %). In terms of educational status, 60 % reported having a post-secondary education. Non-immigrants made up 75 % of the total participants. Nearly one-half of the participants had an average annual income of $80,000 or more, while a very small proportion (7.3 %) reported having no income or earned less than $20,000.

Three-quarters of participants were employed. In terms of living arrangement, 14 % of the participants reported that they live alone. 39 % of respondents were categorized as physically active and 18 % sedentary. Just over one-half of respondents were overweight or obese. 12 % of the respondents were daily smokers and 60 % were regular drinkers (see Table 1).

**Table 1:** Percentage distribution of participants’ characteristics, CCHS 2015-16, Canada

Sociodemographic characteristics	Weighted %	Missing (%)
**Sex**		0.0
Male	49.34	
Female	50.66	
**Ethnicity**		7.6
White	80.55	
Aboriginal	16.36	
Visible minority	3.09	
**Age**		0.0
< 24	17.53	
25–40	23.80	
41–65	40.34	
65+	18.34	
**Marital status**		0.3
Married	46.42	
Common-law	11.36	
Widowed/divorced/separated	12.16	
Single	30.06	
**Educational level**		1.4
Less than secondary school graduation	18.48	
Secondary school graduation, no post-sec education	21.82	
Post-secondary certificate diploma or university	59.71	
**Immigration Status**		3.8
Landed immigrant / non-permanent	25.31	
Non-immigrant (Canadian born)	74.69	
**Living arrangement**		0.5
Living alone	14.41	
Living with others	85.02	
**Working status − 12 months**		11.2
Yes	75.71	
No	24.29	
**Household income**		0.1
No income or less than $20,000	7.34	
$20,000 to $39,999	15.16	
$40,000 to $59,999	15.05	
$60,000 to $79,999	14.19	
$80,000 or more	48.26	
**Physical activity level**		10.0
Active	38.5	
Moderately active	15.0	
Somewhat active	18.7	
Sedentary	17.9	
**Body Mass Index/ BMI**		13.4
Underweight or normal	32.8	
Overweight	31.0	
Obese - Class I, II, III	22.8	
**Smoking status**		0.1
Daily	12.3	
Occasionally	5.0	
Not at all	82.7	
**Alcohol intake**		0.5
Regular drinker	60.1	
Occasional drinker	16.0	
Did not drink in the last 12 months	23.4	

*Note*. ∗Percentages weighted to the Canadian population

**Fig. 1:**
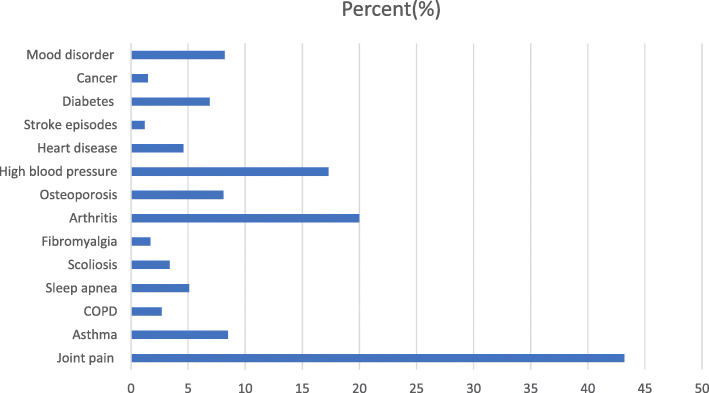
Percentage distribution of study participants by type of self-reported chronic morbidity

As shown in Fig. 1, chronic joint pain is the most frequently reported morbidity followed by arthritis (e.g., osteoarthritis, rheumatoid arthritis, gout) and high blood pressure. The overall prevalence of multimorbidity was 33.3 %. As shown in Table 2, the most common two chronic disease combination was joint pain and arthritis (16 %) followed by joint pain and high blood pressure (10 %).

**Table 2:** Prevalence of the most common two chronic conditions reported, CCHS 2015-16, Canada

Types	Weighted %
Joint pain and arthritis	15.9
Joint pain and high blood pressure	10.1
Joint pain and mood disorder	5.1
Joint pain and diabetes	4.1
Diabetes and high blood pressure	3.8

Table 3 presents the proportion of respondents with multimorbidity according to sociodemographic and lifestyle variables. The table also presents both unadjusted and adjusted odds ratios (with 95 % CIs). Since all variables considered in the bivariate logistic regression.

(i.e. unadjusted) were significantly associated with multimorbidity, all of them were entered into the multivariable logistic regression model (i.e. adjusted).

**Table 3:** Unadjusted and adjusted odds ratios with 95 % confidence intervals for the association of sociodemographic and lifestyle factors with multimorbidity, CCSH, 2015–2016, Canada

	Multimorbidity*			Unadjusted	Adjusted
**Sociodemographic Characteristics**	**Yes (%)**	**No (%)**	**Missing (%)**	**OR(95 % CI)**	**AOR(95 % CI)**
**Sex**			0.0		
Male	30.13	69.87		1	1
Female	36.41	63.59		**1.33(1.27–1.38)**	**1.43(1.32–1.56)**
**Ethnicity**			7.6		
White	36.71	63.29		1	1
Aboriginal	26.80	73.20		**0.59(0.57–0.61)**	**0.87(0.82–0.91)**
Visible minority	15.69	84.31		**0.37(0.34-0 0.40)**	0.97(0.87–1.08)
**Age**			0.0		
< 24	9.43	90.57		1	1
25–40	15.25	84.75		**1.73(1.56–1.92)**	**1.29(1.18–1.43)**
41–65	39.30	60.70		**6.22(5.67–6.82)**	**4.29(3.92–4.70)**
65+	66.39	33.61		**18.97(17.26–20.87)**	**7.75(7.01–8.57)**
**Marital status**			0.3		
Married	37.76	62.24		1	1
Common-law	29.07	70.93		**0.68(0.63–0.72)**	**0.93(0.88–0.99)**
Widowed/Divorced/Separated	56.48	43.52		**2.14(2.01–2.27)**	**1.12 (1.04–1.18)**
Single	18.73	81.27		**0.38(0.36–0.40)**	**0.94(0.88–0.99)**
**Educational level**			1.4		
Less than secondary school graduation	34.48	65.52		1	1
Secondary school graduation	36.03	63.97		**1.07(1.01–1.14)**	0.95(0.89–1.01)
Post-secondary certificate diploma/university	31.75	68.25		**0.88(0.84–0.93)**	**0.92.(0.87–0.97)**
**Immigration Status**			3.8		
Landed immigrant	28.95	71.05		1	1
Non-immigrant (Canadian born)	34.83	65.17		**1.31(1.24–1.39)**	**1.24(1.16–1.32)**
**Working status − 12 months**			11.2		
Yes	26.00	74.00		1	1
No	48.95	51.05		**2.73(2.59–2.87)**	**1.67(1.59–1.74)**
**Living arrangement**			0.6		
Living with family members and others	48.1	51.9		1	1
Living alone	30.8	69.2		**0.47(0.46–0.48)**	**0.94(0.89–0.99)**
**Household income**			0.1		
No income or less than $20,000	44.11	55.89		1	1
$20,000 to $39,999	42.74	57.26		**0.95(0.87–1.03)**	**0.74(0.68–0.80)**
$40,000 to $59,999	38.14	61.86		**0.78(0.72–0.85)**	**0.69(0.65–0.75)**
$60,000 to $79,999	32.69	67.31		**0.62(0.56–0.67)**	**0.66(0.61–0.71)**
$80,000 or more	27.39	72.61		**0.48(0.44–0.52)**	**0.58 (0.54–0.63)**
**Behavioral factors/ lifestyle variables**					
** Physical activity**					
Active	30.36	69.64	10.0	1	1
Moderately active	32.93	67.07		**1.12(1.05–1.20)**	**0.95(0.91–0.99)**
Mild active	35.90	64.10		**1.28(1.21–1.36)**	0.99(0.96–1.04)
Sedentary	46.85	53.15		**2.02(1.90–2.14)**	**1.06(1.01–1.11)**
**Smoking**			0.1		
Daily	41.42	58.58		1	1
Occasionally	27.64	72.36		**0.54(0.48–0.61)**	**0.82(0.73–0.93)**
Not at all	32.45	67.55		**0.68 (0.64–0.72)**	**0.79(0.74–0.84)**
**Body mass Index (BMI)**			13.4		
Underweight or normal	25.76	74.24		1	1
Overweight	34.04	65.96		**1.48(1.41–1.57)**	**1.37(1.32–1.43)**
Obese	48.72	51.28		**2.74(2.58–2.90)**	**2.65(2.54–2.76)**
**Alcohol intake**			0.5		
Regular	31.76	68.24		1	1
Occasional	37.52	62.47		**1.29(1.22–1.37)**	1.00(0.89–1.13)
Not at all	34.22	65.78		**1.12 (1.06–1.18)**	**0.83(0.75–0.91)**
**Sex and smoking interaction**					
Female and daily smoker	-	-		-	1
Female and occasional smoker	-	-		-	0.95(0.80–1.13)
Female and nonsmoker	-	-		-	**0.83(0.76–0.91)**
**Immigration and alcohol intake**					
Non-immigrant and regular alcohol taker	-	-		-	1
Non-immigrant and occasional alcohol taker	-	-		-	**1.23(1.09–1.39)**
Nonimmigrant and non-alcohol taker	-	-		-	**1.51(1.36–1.69)**
Constant					0.156(0.13–0.18)

*F(31, 41,334) = 404.54; Prob > F = 0.0000*

*Note*. ∗ weighted to the Canadian population.; *RC* reference category; The outcome variable is defined as “co-occurrence of two or more (chronic) diseases within a patient; the bold texts in the last two columns indicate the categories are significant (*p* < 0.05)

The last column in Table 3 presents the results of adjusted logistic regression model. Multicollinearity diagnosis among the variables was checked, and none of them had significant collinearity. The odds of multimorbidity decreased by 13 % for aboriginal people (AOR = 0.87; CI:0.82–0.91) compared to white people. Compared to those15-24 years of age, the odds of multimorbidity was higher by 1.29 times (CI:1.18–1.43), 4.29 times (CI:3.92–4.70) and 7.75 times (CI: 7.01–8.57) for the 24–40, 41–65, and 65 + years age groups, respectively.

The likelihood of multimorbidity was 1.12 times higher (CI: 1.10–1.18) for those widowed/divorced/separated, and 6 % lower for those with common law partners compared to married respondents. Post-secondary graduates were less likely to report multimorbidity (AOR = 0.93; CI:0.88–0.99) compared to those who did not graduate high school. Those not employed were 1.67 times more likely to have multimorbidity than their employed counterparts. The odds of experiencing multimorbidity significantly decreased as household income increased. The likelihood of multimorbidity was 6 % lower (AOR = 0.94; CI:0.89–0.99) for respondents who reported living with their family members/others compared to those living alone.

Relative to physically active participants, moderately active participants were 5 % less likely(AOR = 0.95; CI:0.91–0.99) and sedentary participants were 1.06 times more likely, to report multimorbidity compared to physically active respondents. Compared to those normal/underweight, overweight respondents were 1.37 times (CI), and obese respondents 2.65 times (CI), more likely to report multimorbidity.

**Fig. 2:**
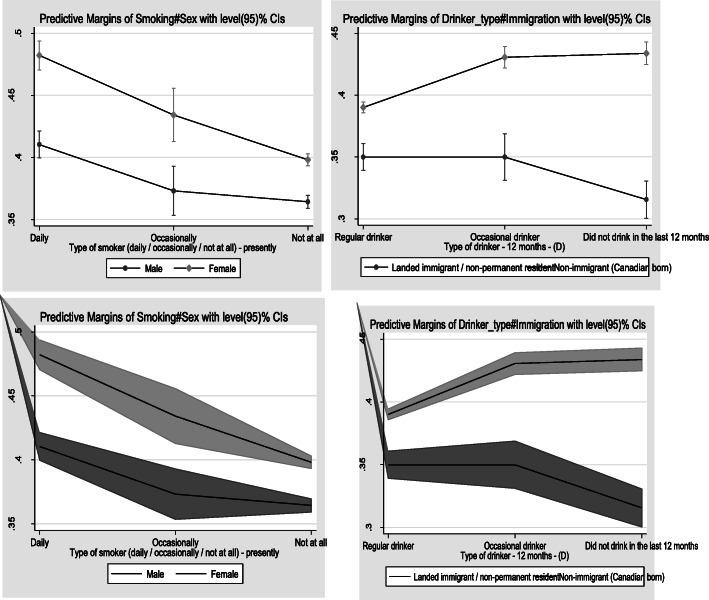
. Interaction plots for sex and smoking (left), and immigration and alcohol intake(right), on additive (top) and multiplicative (bottom) scales

There were two statistically significant interactions; between sex and smoking, and between immigration status and alcohol intake (Fig. 2). Smoking was more strongly associated with multimorbidity in females than males. The relationship between alcohol intake and multimorbidity was dependent upon immigration status. For Canadians, relative to regular drinkers, the likelihood of multimorbidity increased for occasional drinkers and abstainers. Among immigrants, the likelihood of multimorbidity was similar for regular and occasional drinkers but then decreased for those abstaining.

## Discussion

The prime objective of the study was to estimate the prevalence and risk factors of multimorbidity based on the 2015–2016 CCHS data which included 109,659 participants drawn from all Canadian provinces and territories.

Multimorbidity was experienced by 33 % of the study population. In comparison to other Canadian studies, this prevalence is lower than the 42.6 % reported by Nicholsousing et al. (2016)[17] based on a sample of respondents aged 18+. Our estimate is also higher than the prevalence computed by Robert et al. (2016)[6], who reported a national prevalence of 2 + chronic conditions, based on five chronic conditions, to be 12.9 %. The difference in estimates across different studies mainly arises from researchers’ choice of study groups/populations, the number of chronic conditions considered in the study, or both. The difference may also result from the type of data sources used (such as administrative vs. self-reported surveys). For example, the estimated prevalence of multimorbidity by recent work of Allison et al. (2017)[5] was based on people aged 40 + years, considering only five chronic diseases, and using administrative data. The study by Allison and colleagues [5] has very little methodological similarities with ours in terms of target groups, data sources used and number of chronic diseases considered; the only common trait was that both studies covered all provinces of Canada. In some instances, the definition of multimorbidity itself makes comparisons of prevalence difficult. For instance, while almost all Canadian studies defined multimorbidity as two or more conditions, another recent study[16] used an outcome variable of 3 or more chronic conditions and reported a prevalence of multimorbidity in Canada of 14.0 %.

The present study assessed the contribution of four lifestyle factors (physical activity level, alcohol intake, smoking and BMI) on multimorbidity. Findings showed a significant inverse association between physical activity level and the occurrence of multimorbidity. This is consistent with recent findings in high-income, middle-income, and low-income countries which reported higher physical activity was associated with multimorbidity [24, 25]. For instance, Lear et al. (2017)[22], based on a large cohort of 130 843 participants from 17 countries (including four low-income countries and seven middle-income countries), reported significant benefits of dose-dependent associations of all forms of physical activity with reduced mortality and cardiovascular disease risks[22]. However, our findings contrast with some previous studies[13, 32], mainly due to difference in the type of physical activity types considered in the individual studies (i.e., leisure versus general activities)[16]. One commonly known pathway for physical activity is that it helps to control excess body weight, and hence, significantly contributes to a reduction in the development of certain chronic diseases such as blood pressure, heart disease, and cancer [16].

The results of this study also showed a significantly higher likelihood of multimorbidity as participants’ BMI increased, a result consistent with other findings conducted around the world. For instance, in a recent study by Jovic and collogues (2016)[24], the proportion of participants who reported two or more chronic diseases increased with each BMI category in both sexes, reaching the highest values in obese category III. Obesity is often highlighted because it is simultaneously a disease and a risk factor for other chronic diseases, such as hypertension and diabetes[33]. There is a general consensus among researchers and medical practitioners that weight loss or having optimal body weight is usually associated with reductions in the incidence of a number of morbidities, such as diabetes, stroke, and heart disease[34–36].This may partly explain why BMI is a strong predictor of multimorbidity in this study.

Recent studies investigating the association between multimorbidity and alcohol intake have reported inverse relationships, suggesting that moderate alcohol use may have protective effects against some chronic diseases, such as dementia, ischemic heart disease, and stroke[25–27]. In addition, most previous studies have reported a positive association between smoking and multimorbidity[6]. However, most studies have not considered whether health behaviors interact with other variables to impact multimorbidity. Our analyses revealed that the relationship between smoking and multimorbidity was dependent on sex/gender of the respondent, where smoking had a stronger association with multimorbidity among women than men. Similarly, the relationship between alcohol intake and multimorbidity was dependent on immigration status. Additional research is needed to clarify why the association between these two health behaviors and multimorbidity is modified by sex/gender and immigrant status.

Other than the lifestyle/ behavioral factors described above, our results also indicated that multimorbidity was associated with several sociodemographic factors, including age. It is well established from previous studies that older people were more prone to various commonly known chronic diseases such as heart failure and dementia[6, 15, 18].One plausible reason for the high multimorbidity in the older age group could be related to numerous age-related changes in the physiological state of individuals, for example, changes in metabolism, immune response, and organ function[37].

The results of this study also showed white participants to have higher odds of multimorbidity than aboriginal and visible minority participants. This finding agrees with that reported in an American study in which aboriginals had higher prevalence [21]. The finding is inconsistent with some Canadian studies[6, 14, 16] which compared the prevalence of multimorbidity between aboriginal and white population. These studies showed higher prevalence of multimorbidity among aboriginals compared to whites, adjusting for income and other socioeconomic characteristics. The difference might be due to study coverage and some methodological differences described above.

Widowed/divorced/separated respondents had much higher odds of experiencing multimorbidity compared to those married. While the mechanisms of how marital status influences disease outcomes is not clearly known, some studies attribute the lower multimorbidity among those partnered to improved mental health as a result of social support. [38, 39].

We found an inverse association between household income and the prevalence of multimorbidity, indicating that the odds of multimorbidity decreased as income increased. Previous research conducted in Canada and other parts of the world have reported inconsistent results. Some of them reported findings consistent with the present study/ inverse association [6, 15, 32], and others, positive or non-significant associations [40, 41]. Education was also found to be significant predictor of multimorbidity in the present study. Existing evidence suggest that low education level and poor economic condition may combine to increase the likelihood of multimorbidity[20]. In other similar studies[18, 21], those with higher education had significantly fewer reported diseases. Some authors argue that behind socioeconomic gradients in health are a higher prevalence of risky health behaviors among the poor, such as smoking and alcohol consumption, which are posited as the actual cause of socioeconomic gradients in health [16]. However, a behavioral explanation is unlikely in this study, as associations between income and education remained significant, even after controlling for various health behaviors. The mechanisms by which SES affects multimorbidity likely involves multiple interacting material, behavioral, and psychosocial factors occurring throughout the life course[42]. In terms of education, people with higher levels of education may better understand and adhere to the prevention and/or treatment of disease, while those with lower education levels may experience more problems related to self-management on a daily basis [38, 43].

### Strength and limitations

Since the study was based on a representative sample of respondents from all provinces, the findings can be generalized to the Canadian population. The results will contribute to the limited available literature in the field and serve as a reference points for future researchers and policy makers. The findings may also be useful for national level planning, targeting, and monitoring and evaluating health outcomes, especially for the most common chronic diseases. However, this study is not without limitations. First, due to its cross-sectional nature, it is difficult to draw temporal relationship between the various explanatory variables and outcome of interest. Second, the chronic health conditions reported in the present study may differ widely in terms of severity which may somehow bias our outcome variable, as it was constructed from a mere count of self-reported chronic morbidities. The self-report nature of multimorbidity might also impact the accuracy of the measurement of the outcome due to possible inaccuracy and omission of information by participants. Comparison of our findings with other studies was also difficult due to varied measures of lifestyle variables (such as alcohol intake and physical activity). Also, the lifestyle variables included in this study were not exhaustive as some factors (such as nutritional status and diet) were not part of the analysis due to lack of appropriate variable in the data set.

## Conclusion and policy implications

Given the unacceptable high prevalence of multimorbidity among the general population, these findings suggest that policy makers and service providers should give more attention to the lifestyle factors (i.e., the proximate determinants) which impact the health outcome among a significant proportion of the Canadian population (33.3 %). Policy and program efforts that promote health lifestyle (healthy eating, physical activity, management of body weight, intake of alcohol in moderation and avoid smoking) should be a priority concern. Raising awareness among stakeholders, promoting comprehensive primary care systems that support generalist (i.e. doctors who provide services for multiple morbidities) and considering longer consultations for people with multiple conditions could be other strategies to effectively manage multimorbidity in the population.

## Data Availability

The dataset used for this study are made available by Statistics Canada: https://www.canada.ca/en/health-canada/services/food-nutrition/food-nutrition-surveillance/health-nutrition-surveys/canadian-community-health-survey-cchs.html. Administrative permissions were required to access the raw data from this organization. Public access to the database is open upon permission.

## Abbreviations

CCHS: Canadian Community and Health Survey
CI: Confidence Interval
OR: Odds Ratio
SDGs: Sustainable Development Goals
SES: Socio Economic Status
US: United States
WHO: World Health Organization
